# Dental Legislation—Opinions for and against

**Published:** 1884-04

**Authors:** 


### Editorial, Etc.

---

DENTAL LEGISLATION.—When the bill for regulating the practice of dentistry in the State of Maryland, was on its passage during the recent session of the Legislature, the following views were expressed concerning the necessity existing for such a law, which we publish in order to show how men differ in regard to matters with which they are supposed to be wholly conversant:

"Though it is commendable in the dentists of Maryland seeking to elevate their profession by means of legislative enactments, nevertheless it is questionable whether a law virtually ostracising a large number of successful practitioners would be consonant with the sound and equitable laws which have so characterized our Legislatures. The Legislatures of Maryland have been specially marked for opposition to the psssage of any law interfering with a man's private business; unless it is clearly proven to be for the best interest ot the public they have never as yet interferred. Now who are the originators of the proposed law prohibiting a dentist from practicing his profession after he had spent say nine years in acquiring it. Why they are the professors of the dental colleges of this city and practitioners of more than ten years' standing in the profession throughout the State, numbers of whom are now graduates. Does the proposed law emanate from the people? Has the public been imposed upon by quacks in the dental profession? There is no such a thing as quackery in dentistry. Any one can determine for himself whether his teeth fit and fillings give satisfaction. Work performed by dentists is patent upon its face and their mistakes are not susceptible of being covered up six feet beneath the sod. No complaints come from the people but from a class of men financially interested. Now it cannot be denied, to be a successful dentist a mechanical skill is of paramount importance, while a thorough knowledge of chemistry, physiology and anatomy is not to be deprecated, it must be conceded it is of secondary importance to the majority of the profession. Now it is a known fact that the so-called "high-toned" dentists often boast of being unable to put up a set of teeth, as it is not the lucrative part of their business, operative dentistry being their specialty. Yet they propose an examination in the various departments of medicine for the classical dentists before proceeding any farther in the practice of their profession. A dentist who has built up a practice by his skill and industry and has secured to himself the confidence of his patients has vested rights, which to divest him of would be overstepping legislative authority. And any law having this for its object would be a law as pernicious in its effects as one impairing the obligations of contracts, and as one confiscating private property for public or corporate use without just compensation. To pass a law affecting dentists already in the profession would be somewhat analogous to an *ex post facto* law. While it might justly be passed affecting those who are to enter the professions. Laws proposed for regulating the practice of dentistry have heretofore received no favor by the Congress of the United States and the Legislature of Maryland."

To this the following reply was made:

"The above, a communication denouncing the proposition to establish a State Board of Dentistry, which shall have the power to examine all persons desiring to practice that profession and to pass under their qualifications. We believe that those who oppose such a law have a right to be heard, but we differ decidedly with the views expressed, and doubt the accuracy of some of the statements made.

The safety of thc public demands that every one engaged in a profession having for its object the treatment of diseases or bodily ailments should first give proof of his capacity and fitness to deal with such matters. It is impossible for persons unacquainted with a dentist or a doctor to judge without trying him whether he is skilled in his profession, and the experiment may or may not be profitable. A license or diploma from a competent board of examiners would be *prima facie* evidence of the skill of the holder, and we know of no other scheme which will so effectually guard the public against quackery. A man who suffers with the toothache has had misery enough for one life without being placed at the mercy of a dentist whose apprenticeship may have been passed in a blacksmith shop.

We have no desire to discourage those who, with praiseworthy ambition, have deserted the workshop to take up the dentist's forceps. We only ask that they shall prove that they know sufficient of their new profession to practice it intelligently. For this reason we would not approve of any provision in the proposed law declaring that the applicant for a license shall be a graduate of a dental college. In our opinion this would be unnecessary and unjust. But the Board of Examiners should carefully prepare a list of questions which will cover every point that a man should know to practice dentistry intelligently, and the result of an examination on these questions should be the test of his fitness. A dental college diploma might exempt a man from examination, but it should not be made a pre-requisite. This is the rule in examinations for the bar, and it has worked satisfactorily.

The assertion of our correspondent that there is no quackery in dentistry is almost as absurd as his remark that any one can determine for himself whether his teeth need a dentist's care. He is also in error in stating that a law affecting dentists already in practice would be an *ex post facto* law—such a construction can only be placed upon laws relating to crime and its punishment."

